# Hybrid alkali-hydrodynamic disintegration of waste-activated sludge before two-stage anaerobic digestion process

**DOI:** 10.1007/s11356-014-3705-y

**Published:** 2014-10-16

**Authors:** Klaudiusz Grübel, Jan Suschka

**Affiliations:** Faculty of Materials and Environmental Sciences, Institute of Environmental Protection and Engineering, University of Bielsko-Biala, Willowa 2 Str., 43-309 Bielsko-Biala, Poland

**Keywords:** Pre-hydrolysis, Hygienisation, Two-stage anaerobic digestion, Methane production, Hybrid disintegration

## Abstract

The first step of anaerobic digestion, the hydrolysis, is regarded as the rate-limiting step in the degradation of complex organic compounds, such as waste-activated sludge (WAS). The aim of lab-scale experiments was to pre-hydrolyze the sludge by means of low intensive alkaline sludge conditioning before applying hydrodynamic disintegration, as the pre-treatment procedure. Application of both processes as a hybrid disintegration sludge technology resulted in a higher organic matter release (soluble chemical oxygen demand (SCOD)) to the liquid sludge phase compared with the effects of processes conducted separately. The total SCOD after alkalization at 9 pH (pH in the range of 8.96–9.10, SCOD = 600 mg O_2_/L) and after hydrodynamic (SCOD = 1450 mg O_2_/L) disintegration equaled to 2050 mg/L. However, due to the synergistic effect, the obtained SCOD value amounted to 2800 mg/L, which constitutes an additional chemical oxygen demand (COD) dissolution of about 35 %. Similarly, the synergistic effect after alkalization at 10 pH was also obtained. The applied hybrid pre-hydrolysis technology resulted in a disintegration degree of 28–35 %. The experiments aimed at selection of the most appropriate procedures in terms of optimal sludge digestion results, including high organic matter degradation (removal) and high biogas production. The analyzed soft hybrid technology influenced the effectiveness of mesophilic/thermophilic anaerobic digestion in a positive way and ensured the sludge minimization. The adopted pre-treatment technology (alkalization + hydrodynamic cavitation) resulted in 22–27 % higher biogas production and 13–28 % higher biogas yield. After two stages of anaerobic digestion (mesophilic conditions (MAD) + thermophilic anaerobic digestion (TAD)), the highest total solids (TS) reduction amounted to 45.6 % and was received for the following sample at 7 days MAD + 17 days TAD. About 7 % higher TS reduction was noticed compared with the sample after 9 days MAD + 15 days TAD. Similar results were obtained for volatile solids (VS) reduction after two-stage anaerobic digestion. The highest decrease of VS was obtained when the first stage, the mesophilic digestion which lasted 7 days, was followed by thermophilic digestion for 17 days.

## Introduction

Anaerobic digestion (AD) of waste-activated sludge (WAS) is commonly used in the wastewater sludge treatment, and it is an appropriate technique for the treatment of WAS, before final disposal. This technology is employed worldwide as the oldest and most important process for sludge stabilization (Dohányos and Zábranská 2001; Rahmani et al. 2009). Sewage sludge quantities are highly dependent on the level of treatment and effluent quality required, with greater quantities of sewage sludge being produced as effluent quality criteria are tightened (Peces et al. 2013). Anaerobic digestion (AD) consists of four major steps. The first step, the hydrolysis, leads to solubilization of insoluble particulate matter and biological decomposition of organic polymers to monomers or dimers. It is a rate-limiting step of AD, when complex organic material is degraded and most of the biodegradable material is either enclosed in the microbial cell wall or enmeshed in an extracellular polymeric matrix. This contributes to limiting biodegradability of these WAS and limits the volatile solids (VS) reduction to the level of 35–45 % (Bolzonella et al. 2005; Bhattacharya et al. 1996; Coelho et al. 2011).

Currently, most anaerobic digesters are operated under mesophilic conditions (MAD), in the temperature range of 25–40 °C. This digestion process is well understood and requires less heat to sustain the operation. Furthermore, the process is very robust and stable—due to the large diversity of bacteria. However, the need for more efficient sewage sludge stabilization and pathogen reduction has recently gained a particular interest. The application of thermophilic anaerobic digestion (TAD) (45–70 °C) can solve the problem. Studies undertaken by several researchers (Ahn and Forster 2002; Kim et al. 2002; Song et al. 2004) showed that thermophilic systems are capable of treating higher organic loadings and can accelerate biochemical reactions. Furthermore, such systems have a higher specific rate growth of microorganisms and accelerate interspecies hydrogen transfer. The abovementioned TAD advantages result in an increased methanogenesis potential at lower retention times (Zábranská et al. 2000; Nwabanne et al. 2009) compared with the mesophilic systems. Additionally, the yield of microorganisms per unit of substrate for thermophilic systems is also lower. The lower growth yield of thermophilic anaerobes could be due to their increased decay rate, which doubles the number of mesophilic cultures. The cells under thermophilic conditions have a tendency to lyse quickly; it may also be due to their higher energy requirement for maintenance or the specific molecular properties of enzymatic reactions at thermophilic temperatures (Kim et al. 2002; Amani et al. 2010). The main problems with thermophilic WAS digestion (compared with mesophilic digestion) include: energy requirements, higher operating costs, lower process stability, poor sludge dewaterability, and the production of highly polluted supernatant (chronically high propionate, ammonia-nitrogen, and chemical oxygen demand (COD) concentration). All of the factors mentioned above prevent this technique from being widely used and commercialized (de la Rubia et al. 2006; Gianico et al. 2013; Kim et al. 2002; Meabe et al. 2013; Speece et al. 2006; Zábranská et al. 2000; Zupancic and Roš 2003).

The dual-stage thermophilic/mesophilic (TMAD), mesophilic/thermophilic (MTAD) process, or temperature-phased anaerobic digestion (TPAD) has recently gained a significant interest. This technology takes advantage of thermophilic systems in terms of pathogen control and VS reduction and makes use of process optimization due to staging. What is more, the majority of the digestion takes place in the mesophilic stage, so it is still profitable to operate such systems (Han and Dague 1997; Sung and Santha 2003).

Thermal, chemical, biological, and mechanical processes have been studied as possible processes to disintegrate the WAS flocs/microorganisms, which results in biomass break-up, destruction of microbial cell walls, and cytoplasm release. These substances in the liquid and colloidal state became available for anaerobic degradation, enhance the sludge degradation rate (biodegradability), and potentially increase sludge dewaterability after digestion (Braguglia et al. 2011; Carrère et al. 2010; Eskicioglu et al. 2006; Örmeci and Vesilind 2000). Sonification and mechanical disintegration are probably the most often applied mechanical WAS disintegration techniques (Bougier et al. 2005; Lehne et al. 2001; Nickel and Neis 2007). Other techniques, like ball mills have found less attention. The chemical disintegration in turn is probably the simplest process for particulate organic matter hydrolysis. The effects of pH decrease or increase on WAS have been studied by many authors (Kim et al. 2007; Li et al. 2008). Chen et al. (2007) investigated the effect of pH from 4.0 to 11.0 during WAS hydrolysis. They concluded that under alkaline conditions, the VFA’s production was significantly higher than under other conditions. However, there was no methane generation after keeping pH on the level of 10.0 and 11.0. Alkaline conditioning with sodium hydroxide (dosage of 0.2 g/g VS), aided by ultrasonic field (ultrasonic pre-treatment) was also tested (Li et al. 2008). Şahinkaya and Sevimli (2013) achieved sludge disintegration of 24.4 % after using only sodium hydroxide (NaOH) as a disintegrating agent. Kim et al. (2003) conducted alkaline pre-treatment at pH 12 at ambient temperatures. The COD solubilization values after NaOH, potassium hydroxide (KOH), Mg(OH)_2_, and Ca(OH)_2_ addition were 39.8, 36.6, 10.8, and 15.3 %, respectively. Similarly, following treatment at 121 °C for 30 min, NaOH addition resulted in 51.8 % COD solubilization. The values for other agents were as follows: 47.8, 18.3, and 17.1 % for KOH, Mg(OH)_2_, and Ca(OH)_2_, respectively.

Very few studies reported the usage of pre-treatment methods, prior to two-stage digestion. Toreci et al. (2009) tested high-temperature microwave pre-treatment (175 °C) combined with two-stage mesophilic digestion for three different Solid retention times (SRTs; 20, 10, and 5 days). However, the results obtained were somewhat inconclusive. Although microwave pre-treatment alone improved biogas production and VS removal, for all SRT compared with non-pretreated sludge, the dual-stage digestion alone showed greater biogas production and higher VS removal, whereas microwave pre-treatment associated with dual-stage digestion did not show any improvement of VS removal and biogas production (Coelho et al. 2011).

National and international regulations require that sludge shall be stabilized and hygienized before its land application. Hygienization is a process intended to reduce the content of pathogenic microorganisms to the safe level. In contrast to bacteria, viruses in general, are sensitive to heat and are inactivated by increased temperature.

Sewage sludge commonly contains high amounts of human pathogenic bacteria excreted in feces and urine. The most important pathogenic microorganisms are those transmitted by the fecal-oral route and include bacteria, viruses, and parasites. The enteric pathogenic bacterial constituents include *Salmonella* spp., *Listeria* spp., *Escherichia coli* (enterotoxigenic and enteropathogenic variants), *Campylobacter* spp., *Clostridium* spp., and *Yersinia* spp. Fecal microorganisms (pathogenic or non-pathogenic) are present in sewage and transferred to sludge, where their concentration is even higher than in the wastewater (De León and Jenkins 2002; Lasobras et al. 1999; Mandilara et al. 2006; Strauch 1998). Moreover, many microorganisms survive better when they are associated with solids than when they are suspended in water (Chen et al. 2012; Gibbs et al. 1997; Scheuerman et al. 1991; Straub et al. 1992), and at some stage of sludge treatment, the growth of some pathogenic bacteria (for example, *Salmonella*) may be supported. Therefore, both treated sludge and raw sludge are sources of pathogenic microorganisms. The most common sludge process is mesophilic (35 °C) digestion, which stabilizes the solids, produces a combustible gas, but does not create a pathogen-free effluent (that meets the requirements).

Kearny et al. (1993) utilized mesophilic anaerobic digestion to analyze the survival of pathogenic bacteria in animal waste. The group found that viable numbers of *E. coli*, *Salmonella enterica* serovar *Typhimurium*, *Yersinia enterocolitica*, *Listeria monocytogenes*, and *Campylobacter jejuni* were reduced during processing. What is more, indigenous bacterial strains survived better than laboratory strains. *Y. enterocolitica* was the least-resistant species to anaerobic digestion (necessary to inactivate 90 % of the population; 18.2 days), whereas *C. jejuni* was the most resistant (time necessary to inactivate 90 % of the population; 438.6 days). It suggested significant variations in the susceptibility of different bacterial species to disinfection.

The annual number of food poisonings caused by *Salmonella* (etiological factor) is estimated to be between three hundred thousand to even four million, whereas the mortality rate can reach up to30.6 % (Kiessling et al. 2002). Salmonellosis constitutes a major health problem, including an intestinal infection—characterized by diarrhea, fever, and abdominal cramps that often lasts 1 week or longer. Therefore, it is necessary to properly handle natural fertilizers (including sewage sludge), which can also lead to the contamination of the natural environment. After mesophilic anaerobic digestion, a gradual decrease in the *Salmonella* sp. population was observed. Similar results after mesophilic fermentation was observed by Paluszak et al. (2012). Kumar et al. (1999) investigated the survival of some pathogens in anaerobic batch reactors. In this study, *Salmonella* sp. survived up to 10 days at temperatures of 35 °C. The survival of *Salmonella typhi* increased from 20 to 35 days when the solid contents were increased from 9 to 15 %.

Development of an effective anaerobic WAS digestion systems to produce sludge residue safe for agriculture use, supported by low intensive pre-treatment procedure, was the aim of the study presented. For upgrading the digestion process, WAS was initially pre-treated by the application of soft alkaline sludge conditioning in front of a partial hydrodynamic disintegration, as the dual-pre-treatment procedure. The alkaline sludge treatment leads to the partial dissolution or destruction of flocs structure, swelling, and subsequent solubilization of cell walls. It was assumed that the combination of alkaline treatment and hydrodynamic cavitation would increase the soluble chemical oxygen demand (SCOD) value and upgrade the effectiveness of anaerobic sludge digestion in terms of biogas production and sludge minimization. Additionally, the aim of the study was to achieve a microbiologically safe sludge, for agricultural use, according to tightened European requirements. In order to achieve the above assumptions, the digestion was performed in mesophilic (first step, MAD), followed by thermophilic (second step, TAD) conditions. According to US standards (40 CFR part 503), the sludge used for agricultural purposes should be of class A, which means that it does not contain detectible levels of pathogens (fecal coliform < 1000 MPN/g DS or *Salmonella* sp. MPN/4 g DS).

## Materials and methods

The WAS (samples, concentration of suspended solids (SS) on an average of 9.5 g/L) was taken from the secondary settling tanks and used as a research material. The plant—municipal wastewater treatment facility—is located in south of Poland, and the facility is working according to the enhanced biological nutrient removal (EBNR) processes. The amount of treated wastewater is approximately 90,000 m^3^/day. SRT is about 14 days, and the concentration of mixed liquor suspended solids (MLSS) fluctuates between 4.3 and 4.7 g/L.

## Experimental setup

**Fig. 1:**
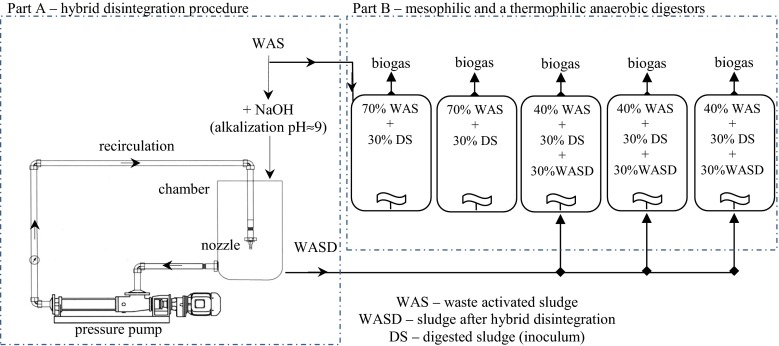
Experimental setup: *part A*, alkalization reactor and hydrodynamic disintegration installation; *part B*, mesophilic and thermophilic anaerobic digestion

A schematic flow diagram of the experimental setup is shown in Fig. 1. The experimental unit consisted of alkalization reactor, hydrodynamic disintegration installation (part A), and mesophilic and thermophilic digestors (part B).

The WAS disintegration was performed in two steps, chemical lysis of microorganism cells, followed by hydrodynamic disintegration, called hybrid disintegration process in the latter part of the paper. For chemical cell’s lysis, 2 M NaOH was used. The pre-treatment reactor was mixed continuously, and simultaneously, the alkali was added in the amount sufficient to keep the selected pH (in the range 7–11) during the entire period of 30 min. In this study, the pH ≈9 was selected as optimum and was used during hybrid disintegration and anaerobic digestion. The amount of 14.4–15.2 mmol NaOH (2 M) per liter of WAS was added to obtain the desired pH value—mentioned above.

**Fig. 2:**
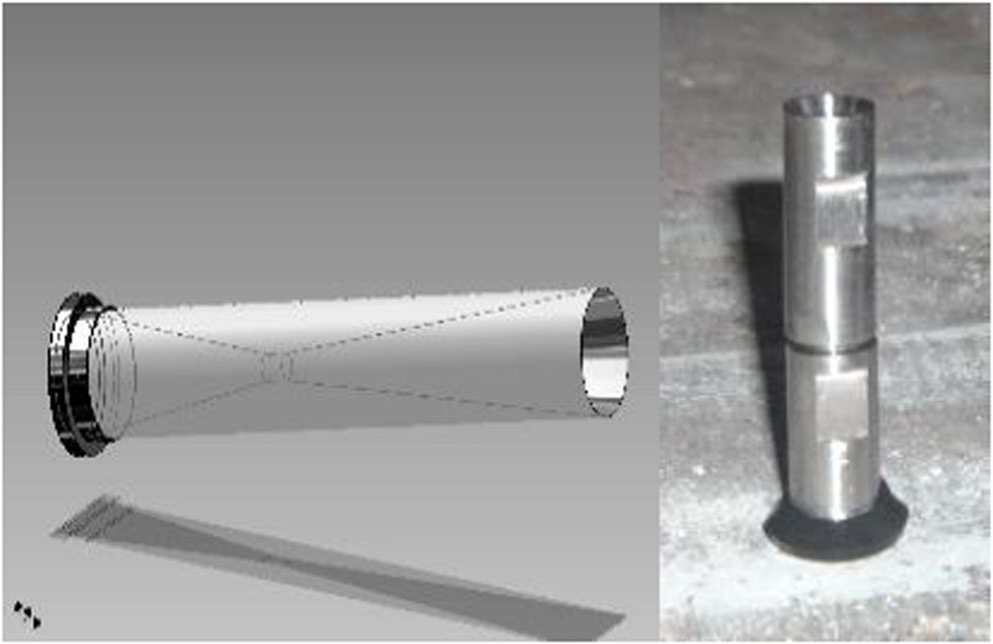
Designed and constructed nozzle

The alkalized WAS samples were directed to the designed 1.2-mm nozzle. The working pressure of the pump was 12 bar, and the corresponding energy consumption of the installation amounted to 0.54 kWh. The 25 L of WAS (the volume used) passed through the nozzle in every 3 min, and the working and the capacity of the installation was 500 L/h. In fact, the disintegration process was continued for 30 min, which corresponded to the 10-fold flow-through cavitation nozzle (recirculation). The designed and constructed cavitation nozzle, based on mathematical models, is presented in Fig. 2. The constructed cavitation nozzle, characterized by a diameter ratio of *β* = *d*_0_/*d*_1_ = 0.30 (*d*_0_, diameter narrowing; *d*_1_, diameter of the inflow), allowed to obtain a cavitation number of *σ* = 0.245, in selected flow conditions. Accordingly, the numerical results of the designed device turned out to be relatively efficient—the calculated pressure loss was Δ*p* = 74.8 kPa, while the net pressure drop (*p*_min_/Δ*p*) was almost five times greater.

## Analytical methods

All chemical analyses were performed for samples before and after each phase of disintegration and during anaerobic digestion.

Total solids (TS), VS, and soluble chemical oxygen demand (SCOD) were determined following the standard methods for examination of water and wastewater procedures 2540G and 5220D, respectively (Rice et al. 2012). VS was measured in triplicate. Firstly, the sample to determine dry solid concentrations, was dried at 105 °C for 24 h. In the next step, the dry solids were incinerated at 550 °C for 2 h. The resulting residue represented the inorganic part of the dry solids. The difference between the total dry solid concentration and the inorganic concentration gave the VS. To analyze the soluble phase, the particulate sludge matter was removed by centrifugation (10 min at 5000 rpm). The resulting supernatant was filtrated through membrane filters (0.45 μm pore size). According to standard methods for examination of water and wastewater, the alkalinity (titration method) was also analyzed.

For colorimetric determinations, a spectrophotometer XION 500 Dr Lange was applied. pH and conductivity measurements were carried out by the application of WTW inoLab Level 2 m, equipped with a SenTix K1 electrode for pH.

The degree of disintegration (DD) was determined according to previously mentioned procedures (Grűbel and Machnicka 2009).

The results presented here were repeated five times and presented as mean values *n* = 5 and standard deviations. The estimator of the highest credibility was used for standard deviation determination (STATISTICA 6.0).

## Two-stages anaerobic WAS digestion

The anaerobic digestion experiments were performed in glass fermenters (3.0 L of working volume). The reactors have been maintained at a constant temperature of 35 ± 1 and 55 ± 1 °C under mesophilic (MAD) and thermophilic (TAD) conditions, respectively. The volume of the biogas produced was measured daily and determined by the liquid displacement method. The first stage of anaerobic digestion, the mesophilic stage (temperature, 35 ± 1 °C), was conducted for 5, 7, and 9 days. After the MAD stage, the new inoculum was added and the temperature was increased to 55 ± 1 °C (thermophilic conditions) and the process lasted for an additional 19, 17, and 15 days.

The fermentation was executed as follows: fermenters 1–2 were fed with WAS (70 % vol.) and inoculum (DS; 30 % vol.) and worked as a control in mesophilic or thermophilic conditions for 24 days, respectively. Fermenters 3–5 were fed with (% vol.): 40 % of WAS; 30 % of DS, and 30 % of WAS after hybrid disintegration (WASD). Fermenters 3, 4, and 5 were undergoing the digestion for 5, 7, and 9 days in mesophilic conditions and 19, 17, and 15 days in thermophilic conditions, respectively (Fig. 1). The aim of the inoculum (DS) added to the mixture was to bring the natural flora of fermentation microorganisms respectively for the mesophilic and thermophilic conditions. The dosage of inoculum has been optimized in previous studies in terms of minimizing the lag phase and decreasing the disturbance of the fermentation process.

The aim of research conducted for various MAD or TAD retention times was to evaluate the effectiveness of two-step anaerobic digestion—in terms of biogas production and biomass reduction. Biogas composition (CH_4_, CO_2_, and O_2_) was analyzed daily, using a gas analyzer Gas Data GFM 416.

## Microbiological analysis

The samples of WAS for microbiological analysis were collected into hermetically sealed containers with a volume of 0.25 L. Prior to sampling, the containers were subjected to a 30-min sterilization in autoclave at a temperature of 121 °C and a pressure of 0.1 MPa.

The following analyses of WAS samples collected before and after the digestion process were performed: *Salmonella* (most probable number—MPN/g dry weight), sulphite-reducing Clostridia (SRC; cfu/g dry weight), *E. coli* (cfu/g dry weight), and somatic coliphages (SOMPCH; pfu/g dry weight). The determinations of microorganisms were made in accordance with Project Routes (2011–2014), Novel processing routes for effective sewage sludge management procedure (Innovative system solutions for municipal sludge treatment and management; grant agreement no. 265156).

The following culture media were used: simple method for *Salmonella* (SMS) Agar, Hektoen Enteric Agar, nutrient Agar, and sulfite polymyxin sulfadiazine (SPS) Agar. In order to verify the taxonomic classification of *Salmonella* sp.*,* the API 20E biochemical and the MUCAP tests were used.

Detection and enumeration of bacteriophages were investigated according to ISO 10705-2: 2000 and proposed European standard.

## Results and discussion

### Hybrid WAS disintegration

According to the given procedure, WAS disintegration was performed in two steps, chemical lysis of microorganisms cells, followed by hydrodynamic disintegration. For chemical treatment (lysis of microorganisms cells), 2 M sodium hydroxide (NaOH) was used. Alkali—NaOH being a monobasic alkali reagent has much higher efficiency of WAS solubilization than dibasic alkali reagents.

NaOH was added to WAS samples in amounts sufficient to maintain a given pH value for 30 min. This is in contrast to many other investigations where the increased of pH was continually maintained for the entire period of digestion. Short-time alkalization applied in this study resulted in cell walls weakening, which what made them more susceptible to lysing processes such as applied hydrodynamic disintegration (second stage of the pre-treatment).

**Fig. 3:**
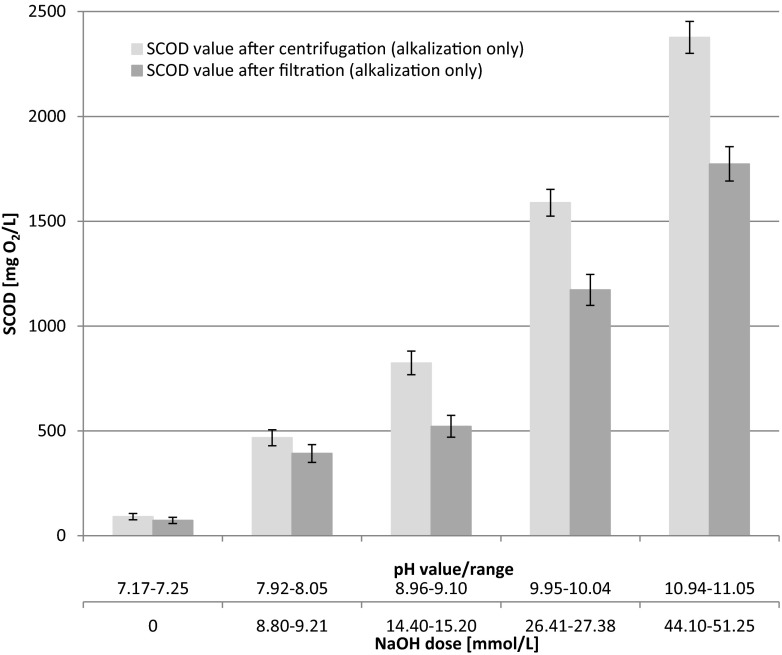
Release of SCOD at different pH values (alkalization) measured as filtrate and centrifugation (mean values + standard deviation)

The results of organic matter solubilization, expressed as soluble chemical oxygen demand (SCOD), are presented in Fig. 3. Release of SCOD at different pH values (alkalization) was measured in samples after filtration (after membrane, 0.45 μm pore size) and centrifugation.

As shown (Fig. 3), alkalization permits achievement of very high solubilization effects. The selection of an appropriate (desired) pH, which depends only on the dosage of NaOH added, is very easy. It is therefore a flexible process and also in full technical scale.

According to Lakshmi et al. (2014), the addition of NaOH to sludge resulted in an effective extracting of EPS, which is the sum of carbohydrate and protein. They tried to determine the efficiency of the sludge pre-treatment and obtained 6.5 % of COD solubilization and 3.8 % of SS reduction.

**Fig. 4:**
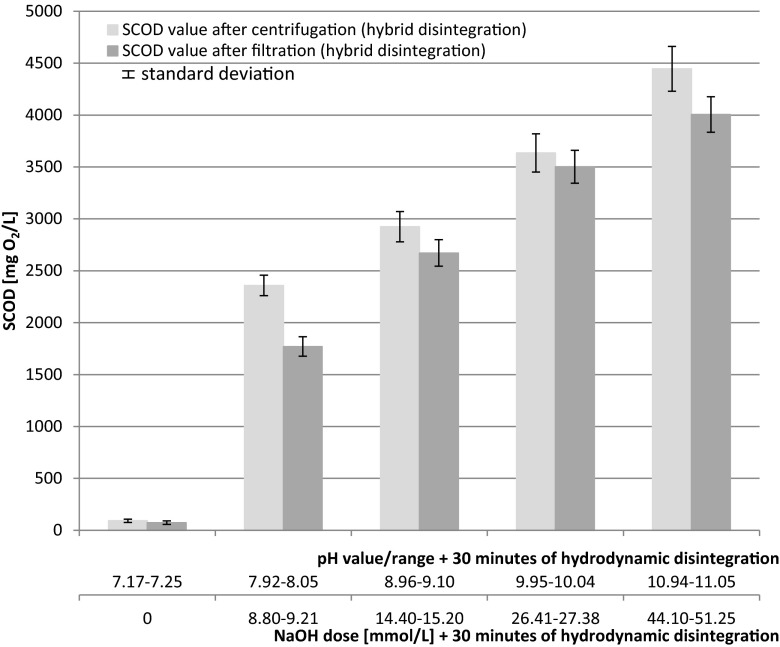
Release of SCOD after the hybrid disintegration (alkalization + hydrodynamic disintegration) measured as filtrate and centrifugation (mean values + standard deviation)

The two-stage (hybrid) disintegration process resulted in a further pronounced increase in the organic matter dissolution (Grűbel et al. 2013; Zhang et al. 2012). As given by Lee and Han (2013), formed hydroxyl radicals generated as cavitation products had a high oxidation potential (*E* = 2.80) and an ability to destroy microorganism’s cells. The solubilization effect of the hybrid technology is presented in Fig. 4.

**Fig. 5:**
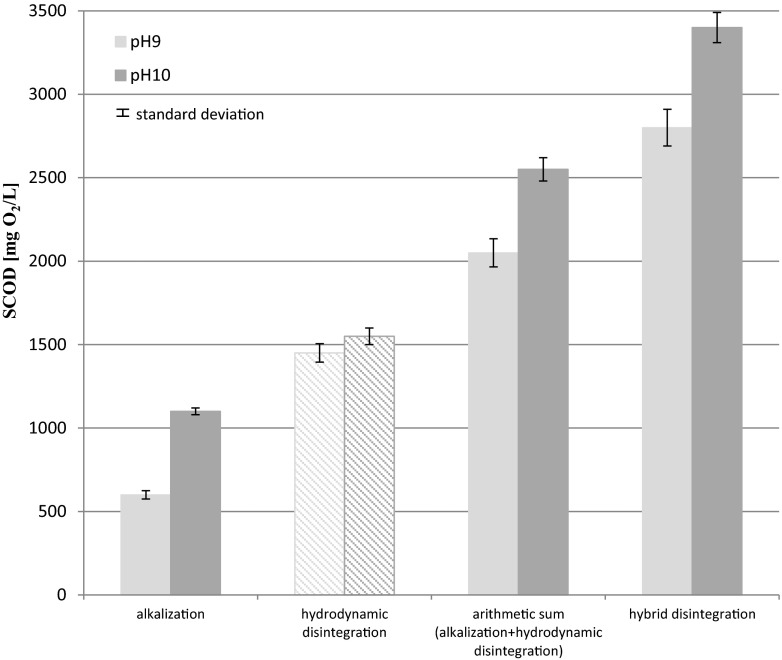
Synergistic effect of hybrid (combined pre-treatment) disintegration technology

The hydrodynamic disintegration of WAS was accelerated by alkalization. The values of SCOD are distinctly higher than the sum of SCOD after alkalization and hydrodynamic disintegration, performed separately. The experiments presented in this study have been limited to the preselected optimal alkalization, i.e., pH 9 and 10. Figure 5 shows SCOD increase as an example and explains the synergistic effect of the hybrid disintegration technology (Fig. 5).

The sum of SCOD after alkalization at 9 pH (in fact pH in the range of 8.96–9.10) equaled to 600 mg/L and after hydrodynamic disintegration amounted to 1450 mg/L; in total, 2050 mg/L. However, due to the synergistic effect, the obtained SCOD value was 2800 mg/L. The latter value constitutes an additional COD increase of about 35 %. Similarly, the synergistic effect for alkalization to 10 pH was also obtained.

Relatively low pH values (9 or 10) were selected as an adequate for effective hydrodynamic disintegration in the second stage. The weakened cell walls make them more susceptible to lysing processes, such as the applied hydrodynamic disintegration. Upgrading distinctly the disintegration effectiveness by a simple and low intensive sludge alkalization (pH in the range of 9 to 10) is of great importance in terms of water–energy nexus. Achieving similar results of hydrodynamic disintegration only would make the process much more energy demanding.

### Anaerobic digestion

After optimization of the pre-treatment method (alkalization + hydrodynamic cavitation), its effectiveness was investigated through carrying out a two-stage anaerobic digestion (mesophilic, followed by thermophilic) of raw and pre-treated sludge. The aim of this part of the research was to maximize biogas production, solids degradation, and pathogen removal. To evaluate efficiency of the entire process with the addition of 30 %WASD to fermenter, different retention times of sludge during the first and second stage of the process were applied.

Considering technological aspects and cost-effectiveness, only partial WAS pre-treatment was adopted. Disintegration of 100 % of the feedstock led to a drastic decrease of the fermentation effectiveness and digested sludge quality. Based on several factors, including high power consumption and additional chemical sludge decomposition, the hydrodynamic disintegration time of 30 min was tentatively chosen for further experiments. The dosage of 30 % vol. of WASD was found (based on multiple repetition of the fermentation process—with varied amounts of disintegrated sludge—different volumes and values of the degree of sludge disintegration (DD)) to be the most favorable and ensured the optimal effectiveness of the anaerobic digestion. Also, the DD was carefully determined on the basis of many tests of the hybrid technology analyzed, combination of alkalization at pH ≈9, followed by hydrodynamic cavitation for 30 min. The applied hybrid pre-hydrolysis resulted in a disintegration degree of 28–35 %. The usage of alkali for disintegration increased the pH of the WASD. However, the dose of WASD was only 30 % of the feedstock total volume and the final mixtures of feedstock directed to anaerobic digestion showed the neutral pH values, which are favorable for methanogens. Thus, it had no effect on the subsequent anaerobic digestion.

The effectiveness of the two-stage anaerobic digestion process was discussed with respect to VS removal and biogas production. Previous experiments have shown that the mesophilic digestion stage, applied as the first stage, should be relatively short, i.e., below 10 days. In order to be able to compare the current results with conventional sewage sludge digestion technologies, the total digestion period was selected to be 24 days. As already described in the methodology, three combinations of mesophilic stage (35 ± 1 °C), i.e., 5, 7, and 9 days and thermophilic stage (55 ± 1 °C), i.e., 19, 17, and 15 days digestion have been investigated.

**Fig. 6:**
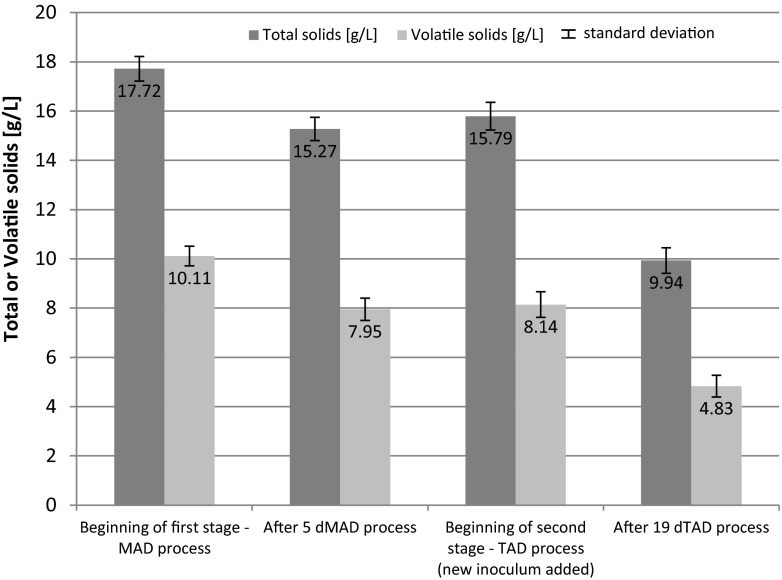
Changes of total and volatile solids during two-stage digestion process—combination of 5 days MAD + 19 days TAD

**Fig. 7:**
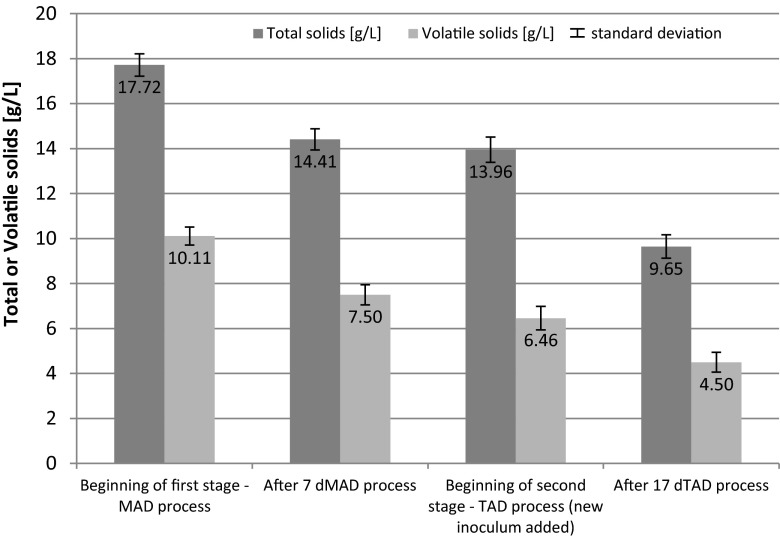
Changes of total and volatile solids during two-stage digestion process—combination of 7 days MAD + 17 days TAD

**Fig. 8:**
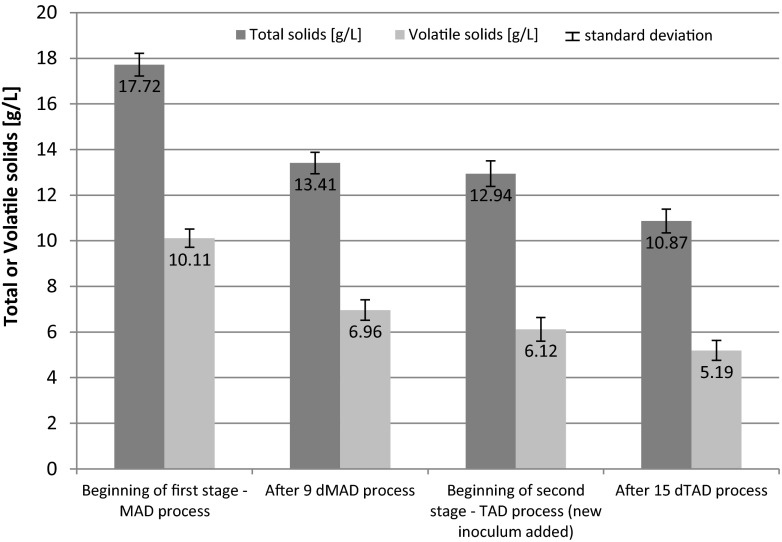
Changes of total and volatile solids during two-stage digestion process—combination of 9 days MAD + 15 days TAD)

Results of VS decomposition for the three combinations are illustrated in Figs. 6, 7, and 8. The data presented are the mean values plus the standard deviations.

The difference between the measured amounts of sludge before and after anaerobic digestion shows the rate of solids degradation. TS and VS were determined before and after, both at the MAD and TAD stages. Determinations of the total and VS before the thermophilic stage were necessary, as results of samples collected, for various determinations after the first digestion stage, have diminished the sludge amount.

The data presented in Fig. 6 shows the concentration of total and VS at the beginning of the first stage of anaerobic digestion and after 5 days of MAD process. TS and VS of digested sludge decreased in the first stage (5 days MAD)—from 17.72 and 10.11 to 15.27 and 7.95 g/l, respectively. After that period of time, the temperature of the process was raised to 55 ± 1 °C and a new inoculum (from thermophilic digestion process) was added. TS and VS of digested sludge after the second thermophilic process (19 days TAD) decreased from 15.79 and 8.14 to 9.94 and 4.83 g/l, respectively. Similar results were obtained for longer periods of mesophilic process, i.e., 7 and 9 days (MAD). The results are presented in Figs. 7 and 8.

**Fig. 9:**
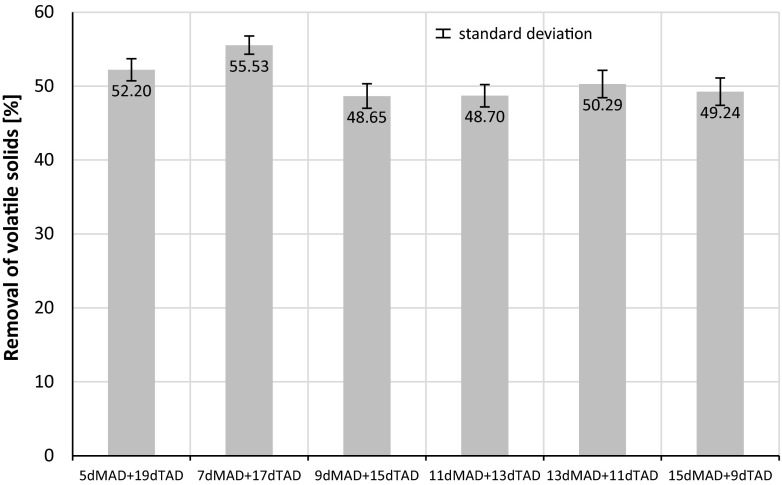
Removal of volatile solids reduction for different duration of the first MAD and second TAD digestion stages (mean values + standard deviation; *MAD* mesophilic anaerobic digestion, *TAD* thermophilic anaerobic digestion)

After the first stage of anaerobic digestion (mesophilic conditions), for the feedstock containing 30 % of sludge disintegrated by hybrid technology, the TS concentration decreased by about 13.8, 18.7, and 24.3 % after 5, 7, and 9 days of MAD, respectively. The highest VS reduction of 31.1 % in the MAD process was achieved for 9 days. After two stages of anaerobic digestion (MAD + TAD), the highest reduction of TS content was achieved for the sample after 7 days MAD + 17 days TAD—45.6 %. About 7 % higher TS reduction was noticed in comparison with the sample after 9 days MAD + 15 days TAD. The similar results were obtained for VS reduction—after a two-stage anaerobic digestion. The highest decrease of VS was obtained when the first stage, the mesophilic digestion, was maintained for 7 days, followed by thermophilic digestion at 17 days (Fig. 9).

Rani et al. (2014) reported that the sono-alkalization pre-treatment of WAS before semi-continuous anaerobic digestion (mesophilic conditions) resulted in the number of percent of COD solubilization and suspended solids reduction after increasing pH value. Rani et al. (2014) used NaOH, KOH, and Ca(OH)_2_ for chemical disintegration. They observed synergistic effect mechanism when sonolysis and alkalization were used. They claimed that the sludge flocs were first disintegrated by hydro-mechanical shear forces, created by ultrasonic irradiation and thus, penetrability of bacteria cell by alkaline was increased. Similar results were reported by Rani et al. (2012).

The obtained results showed the importance of the first mesophilic digestion stage (MAD) duration, in respect to organic matter removal. Prolonging the duration of the MAD stage to 11, 13, and 15 days, however, did not influence the achieved percentage of VS reduction (Fig. 9).

As the organic matter decomposition and MAD period were prolonged, the amount of biogas produced also increased. A further slight biogas production was also observed under thermophilic condition, suggesting that the majority of the organic compounds were converted during the mesophilic stage. The produced biogas and related parameters are summarized in Table 1.

**Table 1:** Results and conditions after MAD process and two-stage MAD + TAD anaerobic digestion processes (mean values ± standard deviation)

	After 5 days of MAD process	After two-stage anaerobic digestion process (5 days MAD + 19 days TAD)	After 7 days of MAD process	After two-stage anaerobic digestion process (7 days MAD + 17 days TAD)	After 9 days of MAD process	After two-stage anaerobic digestion process (9 days MAD + 15 days TAD)
Biogas (cm^3^/L)	2560 ± 85	3946 ± 95	3320 ± 105	4219 ± 103	3474 ± 125	4144 ± 115
CH_4_ (%)	–	63 ± 1	–	64 ± 1	–	62 ± 1
O_2_ (%)	–	1.2 ± 0.05	–	0.8 ± 0.06	–	0.9 ± 0.06
ORP (mv)	−267.8 ± 35	−359.6 ± 25	−286.2 ± 28	−336.4 ± 18	−352.4 ± 15	−380.0 ± 24
pH	7.40 ± 0.2	7.28 ± 0.2	7.66 ± 0.3	7.40 ± 0.2	7.52 ± 0.2	7.45 ± 0.2
Alkalinity (mg CaCO_3_/L)	2665 ± 65	2705 ± 75	2595 ± 45	2735 ± 60	1875 ± 53	2795 ± 95

*MAD* mesophilic anaerobic digestion, *TAD* thermophilic anaerobic digestion

**Fig. 10:**
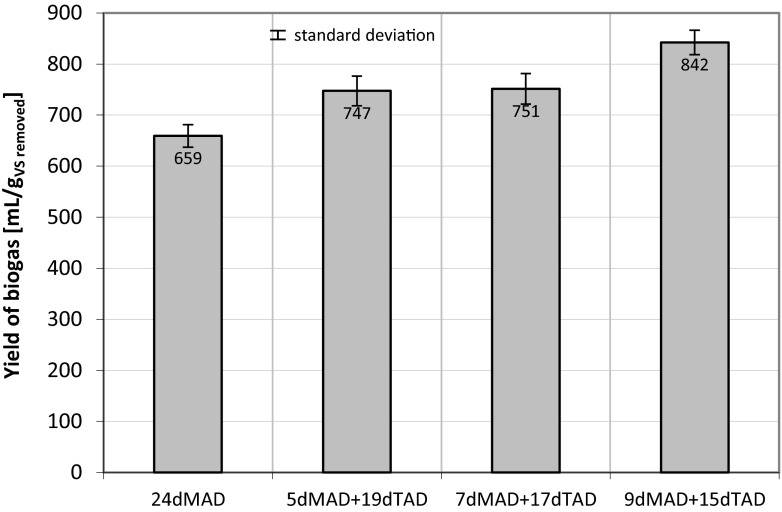
The yield of biogas depending of the time of first MAD and second TAD digestion stages (mean values + standard deviation; *MAD* mesophilic anaerobic digestion, *TAD* thermophilic anaerobic digestion)

It has been noted that, in case of all process conditions applied (different rates of digested sludge or varied disintegration degrees), the content of methane in biogas produced was in the range of 62 to 66 %. Rani et al. (2014) observed an increase of cumulative biogas production after applying sono-alkalization as a WAS pre-treatment, before semi-continuous mesophilic anaerobic digestion (mesophilic conditions). They concluded that addition of NaOH to WAS at pH 10 leads to the best performances in terms of COD solubilization, SS reduction, and biogas production. The biogas yield increased as the length of the MAD process was prolonged (Fig. 10).

The obtained results demonstrate the distinct effect of prolonged time of MAD stage on the biogas yield. Considering the two extreme cases, namely the digestion in one stage of MAD (24 days) and TAD (24 days), the yield of biogas amounted to 659 mL/g_VSremoved_ and 848 mL/g_VSremoved_, respectively. After two stages of anaerobic digestion (MAD + TAD), the highest yield of biogas was achieved for the sample after 9 days MAD + 15 days TAD—842 mL/g_VSremoved_. Compared with the sample after 24 days MAD process, the biogas yield increased by 27.8 % after 9 days MAD + 15 days TAD. Extending the duration of the MAD stage for 11, 13, and 15 days did not influence the achieved biogas yield (data not shown).

### Feasibility of digested sludge agriculture application

In this research work, the influence of digestion process alone and the dual method of pre-treatment (alkalization and hydrodynamic disintegration) and subsequent anaerobic digestion on the microbiological quality of WAS, were taken into consideration. Mean values of biological indicators are presented in Tables 2 and 3.

**Table 2:** The average numbers of microorganism indicators—somatic coliphages (SOMPCH) and most probable number (MPN) of *Salmonella*—before and after mesophilic/thermophilic fermentation process (mean values ± standard deviation)

Sample (% volume in the reactor)	SOMPCH (pfu/g dry weight)			*Salmonella* (MPN/g dry weight)		
	Start of the process	End of the mesophilic process	End of the thermophilic process	Start of the process	End of the mesophilic process	End of the thermophilic process
70 %WAS + 30 %DS (only MAD)	(4.16 ± 0.15)∙10^5^	(1.63 ± 0.18)∙10^4^	–	>11	11	–
40 %WAS + 30 %DS + 30 %WASD (only MAD)	(9.16 ± 0.21)∙10^4^	(7.76 ± 0.24)∙10^3^	–	11	2.9	–
40 %WAS + 30 %DS + 30 %WASD (5 days MAD + 19 days TAD)		(5.23 ± 0.19)∙10^4^	0		2.1	0
40 %WAS + 30 %DS + 30 %WASD (7 days MAD + 17 days TAD)		(4.11 ± 0.23)∙10^4^	0		2.1	0
40 %WAS + 30 %DS + 30 %WASD (9 days MAD + 15 days TAD)		(3.48 ± 0.17)∙10^4^	0		1.5	0

“%” volume of sludge, *WAS* waste-activated sludge, *DS* digested sludge, *WASD* WAS after hybrid disintegration, *MAD* mesophilic anaerobic digestion, *TAD* thermophilic anaerobic digestion, *pfu* plaque-forming unit

**Table 3:** The average numbers of microorganisms indicators—sulphite-reducing Clostridia (SRC) and *Escherichia coli*—before and after mesophilic/thermophilic fermentation process (mean values ± standard deviation)

Sample (% volume in the reactor)	SRC (cfu/g dry weight)			*E. coli* (cfu/g dry weight)		
	Start of the process	End of the mesophilic process	End of the thermophilic process	Start of the process	End of the mesophilic process	End of the thermophilic process
70 %WAS + 30 %DS (only MAD)	(4.25 ± 0.23)∙10^5^	(3.38 ± 0.17)∙10^5^	–	(5.23 ± 0.27)∙10^5^	(5.68 ± 0.25)∙10^3^	–
40 %WAS + 30 %DS + 30 %WASD (only MAD)	(3.43 ± 0.18)∙10^5^	(9.75 ± 0.15)∙10^4^	–	(9.68 ± 0.29)∙10^4^	(3.18 ± 0.21)∙10^3^	–
40 %WAS + 30 %DS + 30 %WASD (5 days MAD + 19 days TAD)		(2.83 ± 0.19)∙10^5^	(2.46 ± 0.24)∙10^2^		(3.54 ± 0.23)∙10^4^	0
40 %WAS + 30 %DS + 30 %WASD (7 days MAD + 17 days TAD)		(2.75 ± 0.21)∙10^5^	(3.25 ± 0.15)∙10^2^		(2.78 ± 0.22)∙10^4^	0
40 %WAS + 30 %DS + 30 %WASD (9 days MAD + 15 days TAD)		(1.43 ± 0.14)∙10^5^	(4.46 ± 0.18)∙10^2^		(1.82 ± 0.26)∙10^4^	0

“%” volume of sludge, *WAS* waste-activated sludge, *DS* digested sludge, *WASD* WAS after hybrid disintegration, *MAD* mesophilic anaerobic digestion, *TAD* thermophilic anaerobic digestion, *cfu* colony-forming unit

One of the goals of the two-step anaerobic digestion process was to improve pathogens removal. The average numbers of SOMPCH and the most probable numbers of *Salmonella* before and after two-stage anaerobic digestion are shown in Table 2.

Bacteriophages included SOMPCH as potential model organisms in determining the influence of sludge treatment on the enteric virus content. Some bacteriophages are structurally similar to human enteroviruses and may be vulnerable to the same inactivating mechanisms. Furthermore, phages are much more easier and inexpensively assayed than enteroviruses. These considerations make bacteriophages attractive system indicators for enteroviruses inactivation. In fact, phages have been proposed as an indicator of viral pollution.

Depending on the length of MAD process, a slight reduction of SOMPCH (from 10.4 to 15.5 %) after mesophilic fermentation was observed (Table 2). Similar results of SOMPCH reduction (8.5 %) after mesophilic fermentation were obtained by Mandilara et al. (2006).

The impact of two-stage anaerobic digestion on the average numbers of sulphite-reducing Clostridia (SRC) and *E. coli* is depicted in Table 3.

Kearny et al. (1993) investigated the efficiency of full-scale anaerobic digestion operated at 28 °C, to remove pathogenic bacteria. *E. coli, S. typhimurium, Y. enterocolitica, L. monocytogenes*, and *C. jejuni* were only partially removed during the applied treatment period.

The total elimination of microbiological contamination (except for SRC; Table 3) was obtained after two-stage anaerobic digestion.

## Conclusions

The pre-treatment of WAS before anaerobic digestion permits acceleration of organic matter decomposition and larger biogas production. The applied two-stage WAS disintegration, based on chemical treatment (alkalization NaOH)—the first step and hydrodynamic cavitation and the second step—was confirmed to be a very attractive technology.

The two-stage pre-treatment process resulted in a significant microorganism cell destruction and organic matter solubilization. Due to the synergistic effect, the second step of the pre-treatment, hydrodynamic disintegration, allowed to receive an extra organic matter solubilization and consequently SCOD values increased above 3500 mg/L.

The main advantage of the hybrid pre-treatment presented include: its relative simplicity, high flexibility (easy and fast adaptation of process conditions to the specific requirements), low chemical, and power consumption. Also, low operating pressure of the hydrodynamic disintegration (12 bar) is of high importance of the technology. The applied technology can be classified as a low intensive, highly effective process of WAS pre-treatment.

The applied two-stage anaerobic digestion—mesophilic plus thermophilic conditions—allows organic matter (VS) reduction above 50 % and a high biogas production, i.e., the yield of 750 mL/gVS_removed_.

Another important advantage includes the production of digested sludge, which is microbiologically safe, and thus can be used for agricultural application.
